# Mr. Harrold, on the Use of Ol. Terebinth. in Burns

**Published:** 1806-02

**Authors:** E. Harrold

**Affiliations:** Cheshunt


### *To the Editors of the Medical and Physical Journal.*

Gentlemen,

I Was pleased at seeing some remarks of Dr. Kentish, on the use of ol. terebinth. in burns, in the 81st Number of your valuable Journal. I do not think that his mode of treating these miserable cases is sufficiently known or properly appreciated; and I must say, that I think every medical man, who wishes to assist his patients in the best and most effectual manner, ought to possess Dr. K's book. Fire is the most powerful stimulus with which we are acquainted. I suppose that most of your readers have read Brown as well as Darwin, and of course will immediately see, that when a powerful stimulus has been applied for a sufficient length of time to produce its proper

---

"A treatise nearly universal in its object, has been particularly desirable, and Dr. Thomas having had opportunities of actually observing the diseases and practice of different countries, but especially those of hot climates, and being conversant with the writings of our best modern authors and teachers, may be considered as well qualified to undertake so important a task.

"We think he has acquitted himself in a manner highly creditable to him, as a man of research and as a practical physician, and that his work deserves to stand high in the catalogue of this kind of compilation."

London Medical Review, Vol. viii. No. 37.

proper or natural effect, and is then suddenly withdrawn, indirect debility is the consequence. They must also be aware that the Brunonian method of preventing or curing indirect debility, is to begin with a stimulant next in power to that which produced the disease, or diseased tendency; and gradually to reduce the excitement, by stimulants less and less powerful, down to the degree of health. This appears to me to be the most simple view of the subject; but this *alone*, is not sufficient to indicate the proper mode of treatment in cases of burns and scalds; for it would lead to the employment of *external* remedies only. It is then farther necessary to consider the skin as an organ of sense, and as sympathising very powerfully with the head, stomach, &c. In cases of burns and scalds (where the injured surface is sufficiently large to affect the whole system) there is manifestly that state of reverse sympathy, so beautifully explained by Darwin; that is, in proportion as the excitement of the surface is plus, that of the heart and arteries is minus; and, unless the excitement is balanced, there will be great danger of death. It is, for this reason, necessary to employ powerful *internal* as well as external stimulants, and to begin with the former. I am sure that practitioners in general do not know how much they are indebted to Dr. Kentish for his happy and successful application and elucidation of these theories; and yet they must all have, at times, seen how extremely untractable burnt wounds are, when the vessels of the part (after having been for some time in a state of indirect debility), by their connection with the system at large, acquire a new and diseased action, producing a fungus difficult to keep within bounds, (especially where a bandage cannot be applied) and little or not at all disposed to cicatrize. A few weeks ago, I was requested to see a child about two years and a half old, who had above one quarter of his surface scalded by boiling water; that is to say, the whole of the right arm to the extremities of the fingers; across the shoulders half way down the scupulæ and to the roots of the hair; the whole of the right cheek and ear; some part of the throat, and almost the whole of the right breast; the axilla and part of the side. Scraped potatoe and linseed oil had been applied, which were not removed without difficulty. The cuticle was entirely off the upper arm and across the shoulders, and large blisters had risen on several other parts of the scalded skin. I immediately gave the child the fourth part of a mixture, containing tinct. opii gtt. XL. and spirit my-

 rist.

rist. ʒvj. (which was repeated at first every fourth and then every sixth hour). I then washed the whole surface with ol. terebinth; at first I used it cold, but perceiving that it occasioned uneasiness, and being at the same time aware that it would be much better warm; it was heated, by putting the phial in a bason of hot water, and it then seemed to occasion no uneasy sensation at all. The wounds were then covered with plasters of ung. rezin flav. softened with ol. terebinth. which also were warmed before they were applied, for these gave pain if applied cold. The first twenty-four hours, spirit and water were given to the child; then wine and wine whey were substituted, and the spirit myrist. was discontinued in the mixture. The dressings were not removed till the next day, when they were renewed exactly as it first; but on the third day almost the whole surface (where the cuticle was removed) was covered with a thick case of pus. Two or three places near the axilla, where it was impossible to prevent friction and to keep the dressings evenly applied, had not suppurated, and these were the last to heal. To these ung. terebinth. was applied, but to the general surface the cerat. lapid. calamin.* On the fourth day, when the dressings were removed, part of the thick case of pus came away in several places, exposing a surface beautifully red, covered with a semi-transparent white film, which proved to be new cuticle. There was a compleat desquamation of the cuticle from the whole of the scalded parts, where i[illegible] was not at first removed, leaving a new but rather irritable cuticle beneath. Finding on this day a copious discharge, I ordered a brisk cathartic, which was occasionally repeated, and which probably accelerates the skinning process by occasioning absorption. In fine, the wounds healed with a rapidity truly astonishing; all except the parts which did not (from friction, &c. as beforementioned) suppurate so soon as the rest. The accident happened on the 14th of last month; my patient has been long since perfectly well, without any scar or inequality of skin remaining. This account will not be compleat without

* It is right to remark, that the cerat. lapid. calamin. is very apt soon to become rancid, when it is an irritating instead of a mild application. To prevent this, it should be frequently made, and not put into a rancid pot. Some of your readers may not know, that a rancid cerate pot may be made perfectly sweet by filling it with small sea coal, pouring upon that as much boiling water as it will then contain, and letting it stand a few days.

without the following remark; that during the first four or five days, a small degree of febrile action was kept up by the internal and external stimuli.

In the eighty-second Number of the Medical and Physical Journal, there are some remarks of Mr. Hall, on the screw tourniquet. I think Mr. Hall has not understood the old screw tourniquet, for he says of two of the rollers of the lower plate, that "they have always been totally unnecessary." I have a screw tourniquet, I suppose twenty years old; it has two rollers on each side at the bottom, and one on each side at the top. The two inner rollers at the bottom, are of use to prevent the friction of the tape against the under plate: a design, of which Mr. Hall does not seem to have been aware; for, in his engraving, the tape is made to pass immediately in contact with the under plate, where the friction must of course be considerable. His design may, however, be easily accomplished, by making two additional rollers at the bottom as well as at the top of the tourniquet. The principal use of this alteration, I conceive, will be in the power of more *instantaneously* relaxing the band after the principal vessels are secured; by which means the smaller vessels which may require a ligature, will be more easily detected by their greater disposition to bleed, from the sudden impetus of the blood.

As every little circumstance in a thing of this sort may be of consequence, I will just remark, that the buckle upon Mr. Hall's tourniquet is not the best for the purpose. The spindle of the buckle upon my old tourniquet, is made much nearer the side to which the band is fastened, and the tongues are of course longer, by which contrivance there is sufficient room for the *points of the fingers* to pass with the band through the buckle.

I am, &c.

*Cheshunt, Dec.* 31, 1805. E. HARROLD.

P. S. I am much obliged by the correct manner in which my drawing of the fracture machine has been executed in your last Number, which I have just received. I think an engraving of the one done in perspective, mentioned in pages 12 and 14, might give a better *general* idea of the thing, (though the different parts are not, and indeed in that way could not, be done by measurement) to persons who may not have an opportunity of seeing the machine itself, which is now at the London Hospital.

pital. In cases of fracture of one thigh only, the other limb may be laid upon the bottom of the other fracture-box, without any splint except the outside thigh splint, (which will be necessary to keep the limb in its place, and to fix the strap from the other outside thigh splint) and passing a bandage a few times round the small of the leg and its support below the button 2, fig. 3. The wool compress for the bottom of the fracture-box should be fitted to it, when the joint A, fig. 3, is bent to a right angle; and excepting at the two extremities, the thread which fastens it to the box should not go *through* the compress, but take hold of the *under side only*. To make the compresses sufficiently soft, the wool should be at first pulled out with great care; and to save expence, after they have been used, the wool may be washed with soap and water perfectly clean, and when dry and combed will be as good as at first.

---

