# Errata

**Published:** 1806-03

**Authors:** 


### ERRATA.

Page 147, l. 14, for *head*, read *heart*.

149, l. 2, for *a small*, read *a smart*.

